# Policy Series: Building Momentum for a New Future in Politics and Aging: Examining Economics, Values, Language, and Care

**DOI:** 10.1093/geroni/igaa057.2365

**Published:** 2020-12-16

**Authors:** Michael Lepore, Jean Accius

## Abstract

Coinciding with the 2020 presidential election, the 75th anniversary of the Gerontological Society of America arrives amid the contentious creation of a new future for politics and aging. Increasing inequality, spreading disinformation, and mounting despotism are escalating threats to constitutional democracy, but at the same time other social changes are promoting the development of a more thoroughly caring, intergenerationally just, and robustly democratic society. At the crux of this societal transformation, relentless political inertia on core aging issues, like the role of government in the care and support of older adults, continues to inhibit meaningful change in federal policy, dampening the potential for older Americans to achieve desired future states, like living well despite advanced age or disability. This session examines major contemporary trends at the intersection of politics and aging in the United States. Papers address the economics and demographics of aging, drawing attention to increasing federal spending on older adults, decreasing availability of caregivers, and geographic clustering of older people; changes in the age of the electorate, intergenerational political values, and the growing politically polarization of American society; the tendency for federal initiatives to fail to support caregivers, for reasons of policy history, policy traits, and mass public features, like the political isolation of informal caregivers; and the role of linguistic and metaphorical practices in shaping our experiences and views of aging. Discussion addresses opportunities for the country to become more age-friendly while also sustaining democratic institutions and national unity.

